# Humean time-reversal symmetry

**DOI:** 10.1007/s11229-023-04247-7

**Published:** 2023-07-17

**Authors:** Cristian López, Michael Esfeld

**Affiliations:** Department of Philosophy, University of Lausanne, 1015 Lausanne, Switzerland

**Keywords:** Humeanism, Symmetry, Time-reversal symmetry, The direction of time, Best system

## Abstract

In this paper, we put forward an alternative interpretation of time-reversal symmetry in philosophy of physics: *Humean* time-reversal symmetry. According to it, time-reversal symmetry is understood as a heuristic, epistemic virtue of the best system, not as a property of the Humean mosaic. One of the consequences of this view is that one of the main arguments against a primitive direction of time is rendered harmless, which paves the way for primitivism about the direction of time.

## Introduction

The problem of the direction of time in physics and philosophy has many facets. Some have taken it as evidence of a clash between a temporally symmetric microphysics and a temporally asymmetric macrophysics (Reichanbach, 1956, Price, 1996, Callender, 1997). Others have rather formulated the problem in terms of whether or not the space–time structure comes equipped with a temporal orientation (Castagnino & Lombardi, 2009; Earman, 1974). From a metaphysical perspective, the problem could more generally be seen as whether the direction of time is a primitive element in our ontology or not. In this line, there are then two possible views—*primitivism* and *reductionism*. Primitivists believe that the directionality of time is a necessary posit in one’s ontology in virtue of its explanatory advantages. It is therefore an irreducible, fundamental feature of the natural world that explains many temporally asymmetric phenomena. Reductionists rather believe that the seeming directionality of time requires an explanation in terms of some non-temporal physical asymmetry to which it can be reduced. In this way, the direction of time is the explanandum, whereas the non-temporal physical asymmetry is the explanans. Reductionism can be seen as either conservative or eliminativist. According to the former, the direction of time is non-fundamental, but real; according to the latter, the direction of time is unreal. The metaphysical map of the different philosophical attitudes towards the direction of time then looks as follows: primitivism holds that the direction of time is real and fundamental; conservative reductionism maintains that it is real, though reducible to a non-temporal physical basis; eliminativist reductionism claims that it is unreal *tout court*.

There is a popular argument in the literature that undermines primitivism about the direction of time—the ‘Time-Reversal Argument’, as we call it (TRA henceforth). Because the laws of physics (or most of them) have the property of being invariant under time reversal, a primitive direction of time is metaphysically unnecessary. Since we ought not to be committed to unnecessary properties, entities or relations, the direction of time is, at least, not primitive. The argument is an instance of a common inference in the field, which takes symmetries to be guides to ontology. In this particular case, time-reversal symmetry as a property of the structure of physical theories entitles us to draw conclusions about time in the ontology. If the inference works and is adopted, then primitivism would indeed be unwarranted.

In this paper, we put forward a Humean construal of time-reversal symmetry, which aims to deflate the relevance of the TRA for the debate on the direction of time. According to our Humean approach, time-reversal symmetry is better to be viewed as a heuristic, epistemic second-order law (or metalaw) in the best system, striving for higher systematicity, unification and simplicity of first-order generalizations.^1^ If time-reversal symmetry is so regarded, the TRA loses much of its persuasive force: it becomes an epistemic virtue of physical theories by contrast to a property of the Humean mosaic. If our construal succeeds, it clears the way to primitivism about the direction of time.

The structure of the paper is as follows. In Sect. 2, we briefly present TRA. In Sect. 3, we offer our Humean view of symmetries and, in particular, of time-reversal symmetry. In Sect. 4, we revisit the TRA argument from our Humean construal as conclusion.

## The time-reversal symmetry argument

Let us start by introducing the TRA. After it, we explain the premises in tandem.

- *P1* If the (fundamental) dynamical laws of physics are time-reversal invariant, then a primitive direction of time is metaphysically unnecessary.
- *P2* We should not posit unnecesary properties in our basic ontology.
- *P3* It happens that (most) laws of physics are time-reversal invariant.
- *P4* A primitive direction of time is then unnecesary.
- *C* We should not posit a primitive direction of time in the basic ontology.

It is easy to see that the structure of the argument is an instance of the so-called ‘the symmetry-to-reality inference’ (see Dasgupta, 2016; also Ismael & van Fraassen, 2003, North, 2009, 2021; Baker, 2010). As Farr (2020a) well notes, it is the same argument that Bertrand Russell used to argue against causality (Russell, 1913).

The conditional in **P1** is an assumption that, though it can be challenged, may have some grip (it is assumed in Mehlberg, 1961; Horwich, 1987; Price, 1996; Arntzenius, 1997; Farr, 2020b; Maudlin, 2002 affirms that it is a popular view in the field). At first sight, it cannot appear evident why a theory’s dynamics should be informative with respect to the structure of time. John Earman suggests that a general motivation for this connection is “the realization that laws of motion cannot be written in the air alone but require the support of various space–time structures” (Earman 1989: 46). Consequently, he formulates two principles working as “adequacy criteria” in a general theory *T* (1989: 46):

SP1: Any dynamical symmetry of T is a space–time symmetry of T.
SP2: Any space–time symmetry of T is a dynamical symmetry of T.

Based on these principles we can postulate a correlation between the dynamics and the space–time structure. The issue then comes down to the sort of relation that holds between a theory’s dynamics, its space–time structure and its symmetries. The correlation can be read in two different ways. Going minimal, one can take the adequacy criteria as heuristic, epistemic principles guiding methodologically the formulation of physical theories: they should be formulated in such a way that the underlying geometry has exactly the right structure to suit the dynamics and its symmetries.^2^ This “adequacy” is expressed through the concordance of the dynamical symmetries to the space–time symmetries. However, as it will be argued later on, an epistemic construal of the principles could diminish the relevance of the TRA to extract an ontological conclusion. One can alternatively propose a stronger reading of the principles: they shed light on an ontic correlation in the world between the laws of nature and the space–time structure. Therefore, if an ontic reading is adopted, then the TRA gains some persuasive force—*P1* already involves some ontological content that may deliver an ontological conclusion.

*P2* is less controversial, since it just expresses an epistemic constraint for the basic ontology. Simplicity is usually an epistemic advice when it comes to detail the minimal set of entities, properties and relations upon which everything else supervenes. In this way, the basic ontology provides the ‘building blocks’ of the rest of the ontology and simplicity advises to only posit the necessary. If a property does not make a difference, or it does not play any explanatory role, why do we then need it? Unnecessary or superfluos properties are not good candidates to enter into the basic ontology.

*P3* is not very controversial either, as least as it stands. Most physical theories are indeed time-reversal symmetric, in the sense in which this expression is frequently understood. In general, time-reversal symmetry is a structure-preserving function that maps solution to solutions of fundamental equations of motions. The transformation is meant to represent the idea of reversing the direction of time by transforming the time coordinate and all the relevant magnitudes that depend on the time coordinate as first derivative (though there may be exceptions to this, see Albert, 2000; Callender, 2000; Peterson, 2015; Lopez, 2019, 2021). Time-reversal invariance is also required to keep invariant physically relevant objects of the theory (e.g., the Hamiltonian, the Lagrangian, the transition probabilities) and to satisfy some interpretative constraints (e.g., to preserve the observational content, to reduce the ‘surplus’ structure of a physical theory, etc.). We will nonetheless come back to this afterwards.

*P4* follows by Modus Ponens from *P1* and *P3*. The conclusion follows from *P4* and *P2*. It is worth mentioning that the TRA can lead us to two alternative conclusions, depending on which kind of reductionism is adopted. For conservative reductionists, the TRA entails that the direction of time is not primitive, since it is not part of the basic ontology; but it can still be real, as part of the derivative (or “emergent”) ontology. For eliminativists, the TRA entails that the direction of time is unreal, since what is real collapses into the basic ontology, and the direction of time does not belong to it.

A common way to resist the argument is to prove that *P3* is false. For instance, collapse theories in non-relativistic quantum mechanics are supposed to be non-time-reversal invariant, and therefore, to support a primitive direction of time (see Arntzenius, 1997: p. 218, Callender, 2000, North, 2011; see Lopez, 2022 for some caveats). In quantum field theory, the CPT theorem states that any Lorentz invariant quantum field theory must be also invariant under the composition of charge conjugation, parity reversal, and time reversal. Since neutral kaons and beta particles decays in weak interactions violate CP, they also violate T. Therefore, it is not true that the fundamental laws of physics are time-reversal invariant (see Maudlin, 2002: p. 267, North, 2011: p. 315; for criticisms see Horwich, 1987, Penrose, 2004). An alternative way to resist the argument is to argue against the conditional in **P1**. For instance, Sklar (1974) rejects the conditional for regarding it unsounded and question-begging; Earman (1974) and Castagnino and Lombardi (2009) move the discussion on the direction of time to general relativity, where the concept of time reversal is either secondary or unapplicable.

We are not concerned here with any of these alternative ways to block the TRA. The point we want to make is that TRA is rendered harmless under a Humean construal of time-reversal symmetry. In this line, we more generally reject the view that takes symmetries (and, in particular, time-reversal symmetry) as guides to ontology. Our aim is to deflate the metaphysical role that time-reversal symmetry plays in physics by arguing that, even though it does play an indispensable role in physics theorizing, it is to be justified epistemically, not ontologically. That is, it is to be viewed as a heuristic, epistemic principle that confers an epistemic gain to physical theories in terms of simplicity, systematicity, and unification of first-order laws, but that it does not confer any metaphysical insight about temporality in the ontology.

## Humean time-reversal symmetry

In this section, we present the Humean view of time-reversal symmetry. We first introduce the concept of physical symmetry in some detail. Next, our Humean view of physical symmetries. Finally, our Humean view of time-reversal symmetry.

### Physical symmetries

Although physical symmetries come in many flavors and shapes (internal vs. external, local vs. global, theoretical vs. observational, geometrical vs. dynamical, and so on), all of them are for the most part *formal* notions that apply to mathematical structures. From a general perspective, physical symmetries are transformations that keep some relevant structure unaltered. In physics, most mathematical structures of interest are sets of differential equations that relate to other mathematical structures (e.g., topological and differential spaces). In consequence, physical symmetries are transformations that preserve the space of solutions of such sets of differential equations. In this precise sense, physical symmetries are said to be structure-preserving functions that map solutions to solutions. This is the *formal* definition of a physical symmetry. In a classical setting and in the group-theorical language, it can be defined as follows (see Olver, 1993: p. 92; see also Belot, 2013).

Formal_def_: Suppose a system $\Delta$ of differential equations involving p independent variables ($x={x}^{1}\dots {x}^{p})$ and q dependent variables ($u={u}^{1}\dots {u}^{q})$. The solutions of $\Delta$ are of the form $u=f(x)$. Let $X={\mathbb{R}}^{p}$, with coordinates $x={x}^{1}\dots {x}^{p}$, be the space representing the independent variables, and let $U={\mathbb{R}}^{q}$, with coordinates $u={u}^{1}\dots {u}^{q}$, represent the dependent variables. A classical symmetry group of the system $\Delta$ will be a local group of transformations, $G$, acting on some open subset $M\subset X\times U$ (kinematically possible fields) in such a way that $G$ transforms solutions of $\Delta$ to other solutions of $\Delta$.

Of course, not any transformation will count as a *physical* symmetry. If this were so, the concept would be trivial and it would always be possible to define a transformation that maps solutions to solutions, augmenting the symmetries of a theory at demand. As Gordon Belot mentions (2013), symmetries are rather hard to come by, so their physical definition should be not too liberal. This in general amounts to imposing further constraints on the formal definition. Some of them can be also purely formal –e.g., for Lie transformations, they must be continuous or smooth; for classical symmetries, the infinitesimal generators must only depend on the independent and dependent variables of the theory, etc. Others can be physical –e.g., Hamiltonian symmetries are required to not only preserve the geometrical structure of the phase space, but also the Hamiltonian. And others can be interpretative –e.g., physical symmetries are required to preserve the observational content of a physical theory (see, for instance, Roberts, 2008; Dasgupta, 2016), or to identify surplus structure (see Redhead, 1975; Dewar, 2019).

We acknowledge that it is an open question in the literature which interpretative constraint is the adequate to provide a satisfactory definition of physical symmetries and we do not intend to settle the discussion here (for in detail discussions of the different views, see Dasgupta, 2016; Wallace, 2019). For practical purposes and mutual understanding, we will say that a physical symmetry is:

Physical symmetry_def_: A structure-preserving transformation acting upon a set of differential equations such that it (a) satisfies the formal definition, (b) keeps invariant physically relevant objects of the theory (e.g., the Hamiltonian, the Lagrangian, etc.), and (c) fulfils some interpretative constraint.

For instance, if (c) refers to the preservation of observational content, a physical symmetry will preserve the observational sentences that an agent would report when asked how things appear in two symmetry-related scenarios. This yields the concept of ‘observational equivalence’ such that two symmetry-related models must be also observationally equivalent.

Time-reversal symmetry is a physical symmetry in the aforementioned sense. In particular, time reversal is commonly understood as a symmetry transformation that intends to formally capture the inversion of the direction of time by transforming the time coordinate plus some dynamically relevant magnitudes. In general, time reversal means the inversion of the direction of motion plus the re-parametrization of the time coordinate (see Wigner, 1932, Gibson and Pollard 1976, among others). Since motion is represented differently across physical theories, the details of the time-reversal transformation will be theory-relative. In the Hamiltonian formulation of classical mechanics, for instance, the main features of the time-reversal transformation stem from an analysis of the physics of the simplest cases. So, the starting point is typically a particle moving on a line in a conservative field force. The state of the particle is given by two variables: the generalized coordinates ${q}_{i}$ and the conjugate momenta ${p}_{i}$. A trajectory in the phase space will be described through a set of functions ${q}_{i}(t), {p}_{i}(t)$, which is given by the Hamiltonian:

$$H=\frac{{p}^{2}}{2m}+V(x)$$

As $V(x)$ is constant and independent of time, it plays no role, and it is generally ignored. In their most general expression, the Hamilton equations follow from the Hamiltonian of the system as function of the ${q}_{i}$ s and ${p}_{i}$ s

$${\dot{p}}_{i}=-\frac{\partial H}{\partial {q}_{i}} ; {\dot{q}}_{i}=\frac{\partial H}{\partial {p}_{i}}$$

In which way can the time-reversal transformation be implemented? The answer mostly depends on what time reversing a classical system means, conceptually. Even though there would be much to say here, the most common answer, and one that is quite easy to grasp, is that of a film played backward. So, by time reversing a classical system we mean to generate a transformation that *retraces* the trajectory of a system. Canonically, this is carried out by a transformation $T$ that reparametrizes the time coordinate, changes the sign of the ${p}_{i}$ s, and leaves the ${q}_{i}$ s unchanged.

$$T:t\to -t ; {p}_{i}\to -{p}_{i} ; {q}_{i}\to {q}_{i}$$

In consequence, $T$ transforms the set of all smooth curves $(q\left(t\right), p\left(t\right))$ through phase space. In consonance with the definition of physical symmetry above, $T$ is a physical symmetry because it also keeps the Hamiltonian invariant:

$${TH(q}_{i}, {p}_{i})={H(q}_{i}, {-p}_{i})$$

That time-reversal symmetry holds in Hamiltonian classical mechanics would mean that it must also fulfil some interpretative constraint. For instance, if two classical physical situations are related by time-reversal symmetry, then they would also be observationally indistinguishable; that is, we could not tell any difference between both situations on the basis of the observational sentences reported in each case.

### Humean physical symmetries

Physical symmetries are everywhere in modern physics. But what are they metaphysically speaking? An analogy to laws can be instructive. The dynamics of physical theories is given by differential equations of motion that stand for laws, which, in addition to some initial condition, allow us to describe and predict the behavior of physical systems. But what are the laws, metaphysically speaking? The philosophical landscape, as it is well known, is rather leafy. Some have thought that such laws *govern* phenomena through necessitation relations between universals (Armstrong, 1983); others that laws are primitive posits in one’s ontology that *produce* physical states (Maudlin, 2007); others that laws *emerge* from dispositional properties (Bird, 2007); others that laws *reduce* to non-modal regularities of local facts (Lewis, 1986). Others have even argued that there are not laws at all, at least as commonly understood (Cartwright, 1983). When we ask what laws are metaphysically, we are asking which place they occupy in one’s ontology.

We can raise analogous metaphysical concerns with respect to physical symmetries (see French, 2014). We do not mean that the metaphysical debate about laws of nature can be translated term-to-term into the debate on the metaphysics of symmetries, but there are some analogies that can be insightful. To begin, philosophical positions may be divided into two camps –those who think that symmetries are *real*, and those who think that they are *epistemic* (see, for instance, Brading & Castellani, 2007; Livanios, 2010). Although from a general viewpoint the division may shed some light on the alternatives, it might be a bit coarse grained when we pay attention to the details. When we hold that symmetries are epistemic, do we mean that they are just conventions and that we might sooner or later be dispensed with them? Or when it is defended that symmetries are real, does it mean that they are aspects of reality, or that they can assist us to discover reality? Are they part of the fundamental or of the derivative ontology?

Humeanism about physical symmetries is a metaphysical view that gives an answer to the problem of which place physical symmetries occupy in one’s ontology.^3^ Humeanism in metaphysics of science (Lewis, 1986, 1994; Loewer, 1996, Cohen & Callender, 2009) is a deflationary view on lawhood and modality, which is committed to two theses. First, there is not *de re* modality in the Humean Mosaic. The Mosaic only contains contingent regularities of local matter of facts (Lewis, 1986: p. ix). Second, modal and nomic features of the universe supervene upon non-nomic facts in the Humean Mosaic. Humean structuralists would add that intrinsic and categorical properties also supervene on a micro-physicalist base (Esfeld, 2014; Lyre, 2010). Since the Humean Mosaic is the primitive (or basic) ontology of the natural world, the metaphysical question to raise is *whether* symmetries are part or aspects of the Humean mosaic.

It was mentioned in passing before that the concept of physical symmetry appears mainly in dynamical contexts, first and foremost as a *property* of differential equations and their space of solutions. Since the concept of physical symmetry and that of law of nature are closely related, it is convenient to start with the latter to shed light on the former. For Humeanism, the Mosaic does not contain nomic, modal facts, but it just exhibits contingent regularities to which laws of nature reduce. The Humean regularist view of laws is frequently complemented with the best system approach to lawhood. Frank Ramsey (1978: §12 [1928]) was probably one of the first defenders of the best system approach: knowledge should be systematized in axiomatic, simple systems. Lewis puts it as following:

“the best system is one that strikes as good a balance as truth will allow between simplicity and strength (…) A regularity is a law iff it is a theorem of the best system” (Lewis 1994: p. 478).

Conforming to this, laws are first and foremost generalizations about non-nomic facts in the mosaic under a strict competitive system –only those that achieve certain optimal balance between simplicity and informativeness will be promoted to theorems of the best system, that is, to genuine laws. The main aim of formulating physical theories is then to optimally systematize our knowledge of the natural regularities that we have got to know thus far.

In contemporary Humeanism, laws of nature thus acquire a two-fold nature. On the one hand, they are propositions (generalizations) that express actual regularities. They are not mere tools, nor fictions, but they do have a grip on what the world is like—they supervene upon the mosaic. On the other hand, the evaluation of generalizations in terms of laws of nature is primordially epistemic, that is, the issue is whether they contribute to both the informativeness and the simplification of our knowledge of regularities (see Loewer, 1996). This means that there will be some properties of laws of nature that do not aim to directly capture any regularity but convey these non-empirical virtues.

How would physical symmetries fit in this framework? The metaphysical question is whether physical symmetries are part of the basic ontology (i.e., the Mosaic). It is important to note that a positive answer to this might already be a red flag. If physical symmetries are first and foremost properties of the differential equations of motion and their space of solutions, and the Humean Mosaic does not contain nomic, modal facts, physical symmetries could then hardly be properties of anything in the mosaic. This does not per se mean that physical symmetries are then unreal; they can still be grounded in the regularities of the mosaic, having a supervenient nature. That is, symmetries can be “real” (as part of the ontology), but not primitive or fundamental (as features of the Humean Mosaic). However, more importantly, physical symmetries directly refer to the *first-order generalizations* of local facts in the Humean Mosaic. This suggests that they are primarily grounded in the formal relations defined over generalizations of facts of the mosaic. It means that physical symmetries are only indirectly related to first-order facts in the Mosaic, but they are directly related to regularities in the first-order generalizations of the regularities in the Mosaic. This goes along with Eugene Wigner’s view, conforming to which symmetry principles “provide a structure or coherence to the laws of nature” (Wigner, 1964; see also Brown, 2005: p. 147).

This may resemble Marc Lange’s view of symmetries (Lange, 2007, 2009, 2011), where symmetries are second-order laws (or metalaws), which aim to capture regularities not among first-order facts, but among laws themselves. Yet, Lange conceives of this relation in terms of “government”—symmetries are high-order laws of nature that *govern* low-order laws. In analogy with Lange’s construal of laws, symmetries exhibit nomic stability and counterfactual robustness (modal force), which makes Lange’s view at odds with a Humean account. In our view, symmetries are metalaws in the sense that they are “laws of the laws”, but they do not govern them, but perform a heuristic, epistemic role in the simplification, systematization, and unification of the true generalizations within the best system. In this line, symmetries are precisely the kind of theoretical resources that allow best systems to substantially boost their main epistemic virtues. To the same extent to which our knowledge of the natural world benefits from building generalizations about phenomena, it benefits from imposing symmetries upon how such generalizations must be formulated. In a nutshell, physical symmetries can be viewed as second-order generalizations of first-order generalizations about first-order facts in the Humean Mosaic.

Our view may also resemble Michael Hicks’ Humean view of symmetries (Hicks, 2019). Though we do agree on the main points of his proposal, we disagree with him on relevant aspects. To begin, Hicks claims that symmetry principles “provide a constraint on first-order facts” (Hicks, 2019: p. 1288). We rather hold that symmetry principles provide a constraint on first-order *generalizations* of first order facts. Take Hicks’ example: “in addition to giving us quite general information about what properties and relations are (and are not) instantiated in the world, symmetry principles tell us when two isolated subsystems will behave in the same way, despite having different connections to the rest of the world” (Hicks, 2019: p. 1289). Hicks’ view would be right if there were isolated subsystems. But isolated subsystems are typically idealizations appearing in the laws, that is, in generalizations on first-order facts. In this sense, if symmetry principles constrain the behavior of idealized objects appearing in lawlike generalization, it is reasonable to think of them not as referring to first-order facts, but to the laws themselves (and their elements consequently).

Secondly, Hicks seems to suggest that symmetry principles can give us information about the properties of the world, licensing an inference from the symmetry to the ontology. For instance, it could be argued that in non-relativistic quantum mechanics the Casimir operators of the Galilean Group give us information about the objective properties of the quantum world. Since non-relativistic quantum mechanics is Galilean invariant and the Casimir operators select the Galilean-invariant set of definite-valued observables that commute with all the generators of the group, such operators could give us information about the invariant properties of the theory. In particular, the mass operator, the squared-spin operator, and the internal energy operator (the Casimir operators) would represent the invariant, ‘natural’ properties in the ontology that Hicks wants to read off from the symmetries of the theory. Notwithstanding how much appealing this view may be, Casimir operators are just mathematical entities acting upon complex-valued state-spaces. Why should they represent real properties? Relying on symmetry principles does not help that much since symmetry transformations also act at the level of mathematical entities. Without an additional premise that pins down certain mathematical entities to the ontology (while not others), Hicks’ view risks to fall into what has been called “naïve realism about operators” (Daumer et al., 1996). In our view, it is clear that symmetry principles refer to operators that form the *dynamical structure* of a physical theory, describing change in a simple and informative way. That is, symmetry principles refer to the elements of the dynamical laws, not to first-order facts.

In the light of the previous paragraph, a comment is in order. It might be argued that our view is too strong since Humeans would almost never be justified to draw inferences from the dynamical structure or the geometrical background of our theories to the ontology (e.g., the nature of space and time). This could be especially problematic as our view would collapse into radical forms of Humeanism (Esfeld & Deckert, 2020; Hugget, 2006), where there is a clear-cut distinction between representational resources in our theories to explain phenomena (e.g., a dynamics and geometry), and ontological posits (e.g., space and time). Of course, we acknowledge that there is variety among Humeans and that some of them would well disagree with our interpretations of symmetries (and time-reversal symmetry in particular). However, we uphold that the case we want to make in this paper is independent from radical forms of Humeanism. What we are arguing for is that *symmetry* principles, and time-reversal *symmetry* in particular, are not good guides to delve into the Humean mosaic. In our construal of symmetries, we place them at the level of second-order generalizations, contributing to build up good physical theories. The burden of the proof is then on those that think differently. We remain silent about whether Humeans may have at disposal others means to read off the geometrical structure of space and time from the systematization of regularities.

To summarize our view, the three crucial features of physical symmetries as second-order generalizations are

- simplicity,
- systematicity,
- unification.

By *simplicity*, we mean that by imposing physical symmetries (e.g., space–time symmetries as time translation, space translation or rotations), a simpler formulation of first-order generalizations can be achieved. In this sense, simplicity is a synonymous of ‘reduction’—we *reduce* the amount of conceptual raw material to capture a relevant regularity. This concept is closely related to a known feature of physical symmetries—they reveal ‘surplus structures’ in physical theories (Dewar, 2019; Redhead, 1975). A common example is space-translation invariance in Newtonian mechanics, which suggests that *absolute* positions are superfluous. Hence, physical symmetries may guide us to simpler formulations of a theory, in the sense of formulations that require less structure to account for phenomena.

Simplicity can be also assessed in terms of the sort of first-order generalizations that make it to the best system. Suppose that we formulate a set of generalizations that are not invariant under spatial rotation. It means that we need to give a generalization (a law) for each possible direction in space. So, the set of first-order generalizations that is required to describe the behavior of a physical system will involve different laws depending on how the system is spatially oriented. But suppose that, after some approximations and idealizations, we obtain a rotation-invariant formulation where the behavior of the system is described through a unique first-order generalization that is valid in all directions (rotation invariance means just that). The latter formulation will be simpler than the former (see Rosen, 2008: pp. 125–127). From a Humean view, it will thereby be a better candidate for a law of nature.

By *systematicity*, we mean that physical symmetries allow us to discover new connections between first-order generalizations (and physical theories as wholes) as well as new, unknown solutions from known solutions. For instance, the Curie-Post Principle (see Post, 1971; Redhead, 1975) establishes that a successor theory cannot be more symmetric than the well-confirmed part of the previous theory, given some appropriate conditions. For instance, electrostatic is scale invariant, while classical electromagnetism with moving charges described by the full Maxwell’s equations is not (for details, see Redhead, 1975: 84, pp. 103–104). Physical symmetries then help physicists find inter-theory connections and correspondences between well-confirmed parts of physical theories.

Finally, simplicity and systematicity lead to *unification*. The paradigmatic case is probably the road to the unification of quantum electrodynamics, quantum chromodynamics, and quantum flavor dynamics in the Grand Unified Theory. Although there are different models in race, symmetries have played a central role in all of them. For instance, the Georgi–Glashow model (proposed by Howard Georgy and Sheldon Glashow in 1974) is the simplest model that combines gauge groups $SU(3)\times SU(2)\times U(1)$ into a simple gauge group, $SU(5)$, unifying, for instance, leptons and quarks in the same particle multiplet.

All three features bring to light the epistemic and heuristic role of physical symmetries in Humeanism. First-order generalizations about regularities *epistemically* benefit from adopting symmetric formulations; otherwise, a much more cumbersome complex system of generalizations would be required to explain even basic phenomena. In contemporary Humeanism, such complexity can be a symptom that such generalizations are not genuine laws in the best system, so we are motivated to find better formulations. Physical symmetries just do that work. In some sense, physical symmetries foster “economy of thought”: one can explain the same (or more) with less.

To sum up, Humean symmetries can be defined as follows.

Humean Symmetries_def_: Physical symmetries are second-order generalizations (i.e., metalaws) that strive for the best simplification, systematization, and unification of the first-order generalizations (i.e., laws) of the regularities of local facts in the Humean Mosaic. Physical symmetries are real to the extent to which they are about first-order generalizations grounded in the regularities, but they are not part of the entities, properties, or relations that make up the Humean Mosaic.

### Humean time-reversal symmetry

Humean time-reversal symmetry is hence just an instance of Humeanism about symmetries as introduced above. That is, it is a physical symmetry that primarily strives for simplification, systematization, and unification of the first-order generalizations in the best systems. As such, it is a mistake to interpret it as a property of the Humean Mosaic (i.e., whether the Humean Mosaic is symmetric under time reversal), but it should rather be viewed as a property of the best system. This move opens the way to primitivism about the direction of time.

To begin, time-reversal invariance means that a time-reversal symmetric *formulation* of the first-order generalizations can be given. It means that when formulating the laws of physics, it is not necessary to specify a direction of time. This property of the formulation of a physical theory must not be mistaken with a property in the ontology—a time-reversal formulation of the first-order generalizations obtained out of the regularities in *this* Humean Mosaic does not mean that they are first-order generalizations that would be also obtained out of the time reversed regularities in a time-reversed Humean Mosaic. To establish our argument, it is important to briefly explain two views of symmetry transformations—the passive and the active view. Even though they are thought of as equivalent (and for all practical purposes it might well be so), they possess some noteworthy differences that are relevant for time-reversal symmetry.

According to the *passive view*, a symmetry transformation $S$ relates the *descriptions* of a physical situation $P$ by two different observers, $O$ and $\overline{O }$. Such descriptions are frequently given by assigning a certain state to $P$ plus some evolution law $L$ (i.e., an equation of motion). Thus, $S$ ultimately equals to a function that yields the translation of the description of $P$ by $O$ into the description of $P$ by $\overline{O }$. Thus, $S:O\to \overline{O }$ entails $S:{L}_{O}\to {L}_{\overline{O} }$, where ${L}_{O}$ is the description of $P$ in terms of the evolution laws that $O$ formulates, whereas ${L}_{\overline{O} }$ is the description of $P$ that $\overline{O }$ formulates. It is worth stressing that a passive view of $S$ brings about two different evolution laws, where the physical symmetry *is* the equivalence of equations of motion in two symmetry-related reference frames, $S{L}_{O}={L}_{\overline{O} }={L}_{O}$.

According to the *active view*, a symmetry transformation $S$ does not relate two different observers, but two physical situations, $P$ and$\overline{P }$. Hence, $S$ transforms $S:O\to O$,$S:{L}_{O}\to {L}_{\overline{O} }$, but$S:P\to \overline{P }$. That is, there is only one observer that describes two different physical situations: $P$ through ${L}_{O}$ and $\overline{P }$ through${L}_{\overline{O} }$. The view deserves the name ‘active’ because now $S$ changes the physical situation itself, generating a new one related by$S$. For instance, in the case of the rotation symmetry, $\overline{P }$ is generated by rotating the measurement apparatuses that $O$ uses to make experiments in$P$. In contrast to the passive view, a symmetry transformation compares the results of experimental outcomes, rather than the preservation of some structure at the descriptive level. In this precise sense, $P$ and $\overline{P }$ can be considered equivalent. For instance, in standard quantum mechanics, a symmetry transformation from an active view can be stated in terms of preservation of transition probabilities, $\left|\langle \psi |\varphi \rangle \right|=\left|\langle S\psi |S\varphi \rangle \right|$.

What is peculiar about time-reversal symmetry is that we cannot make experiments on $\overline{P }$. So, an active view of time-reversal symmetry fails to exist, since it is non-realizable. This is well-noted by Fonda and Ghirardi (1970: p. 43): active views can only exist when the physical states realizable by $O$ can be also realizable (by the same observer, or by a symmetry-related observer, $\overline{O }$).^4^ But if this is not possible, the passive view is the only alternative. It is blatant that we do not know what $\overline{P }$ would look like, since we cannot reverse in time the regularities that obtain in the actual Humean Mosaic. But, in addition, it would be a mistake, from a Humean perspective, to infer features of a hypothetical time-reversed Mosaic on the basis of a time-reversal symmetric formulation of the laws of the actual Humean Mosaic.

For the sake of clarity, let us suppose the facts and regularities that obtain in the Humean Mosaic $$w$$ (our universe). Upon it, a set of first-order generalizations, $${L}_{w}$$, supervenes (our best physical theories). Such a set specifies the laws of $$w$$, which are the simplest and most informative generalizations of the $$w$$-regularities. Suppose now that such a set is given a time-reversal-symmetric formulation. This means that we can formulate $${L}_{w}$$ either with $$t$$ or $$-t$$. That is, we do not need to specify in our *description* of the $$w$$-regularities a direction of time. Conforming to Humean standards for theory assessment, $${L}_{w}$$ performs better in some non-empirical virtues (e.g., simplicity) than an alternative set of first-order generalizations that is non-time-reversal symmetric. However, this does not mean that the time-reversed formulation of $${L}_{w}$$ (say, $${TL}_{w})$$ describes the regularities that would be obtained in a time-reversed version of $$w$$ (say, $$Tw)$$. Under the passive view of time reversal, $${L}_{w}$$ and $${TL}_{w}$$ are alternative descriptions of the *same* Humean Mosaic since they supervene upon the same regularities in the Humean Mosaic. What is an equivalence in the descriptions must not be conflated with an equivalence in the ontology.

In fact, we do not know how $Tw$ would look, nor can we have access to it through the laws obtained out of $w$. Neither are we entitled to infer from ${TL}_{w}$ the features of $Tw$. Humeanism about time-reversal symmetry gets the facts the other way around –${L}_{w}$ supervenes upon $w$, so there is no reason to assume that by giving a time-reversal symmetric formulation of ${L}_{w}$ (${TL}_{w}$) we are obtaining the laws that would supervene upon $Tw$. The reason is simple—$Tw$ and $w$ differ metaphysically in the direction of time, so we cannot be sure that the first-order generalizations that supervene upon the $w$-regularities would be equivalent to those that would supervene upon the $Tw$-regularities, regardless how we may have access to them.

Let us put the point a bit differently. The question about time-reversal invariant laws is the question about whether the laws that supervene on this Humean Mosaic can be formulated in a time-reversal invariant way. This is a theoretical, mathematical question. The question of whether there might be an alternative time-reversed Humean Mosaic is however metaphysical, not theoretical-mathematical. The problem is that laws supervening on the actual Humean Mosaic do not give us *always* access to a different Humean Mosaic—e.g., a hypothetical time-reversed one –, on which a different best system, which we do not know and do not have any means to know, would supervene. Hence, why should we assume that an epistemic virtue of the physical laws that supervene on *this* Humean Mosaic is a reliable way to metaphysicaly investigate alternative Humean Mosaics (such as, for instance, a time-reversed one)? Here, again, there is a conflation between ontological and theoretical-mathematical issues: physics cannot help us in the metaphysical exploration—a genuine time-reversed Humean Mosaic might be radically different from our Humean Mosaic, with regularities (if any) unsuitable for our physics. This argument supports and explains, for instance, Tim Maudlin’s well-guided intuition about time reversal (Maudlin, 2002, 2007): there might not even be any physical systems that are living organisms or creatures with mental states in time-reversed worlds. For, what would be an anatomic system functioning backward, or cellular division happening in time reversion? Our view of Humean time-reversal symmetry makes this clear since to even conceive the metaphysical possibility of such time-reversed scenarios is to suppose more modal information than could be obtained from the best system and the regularities upon which the best system supervenes.

It can be argued that such an inference is nonetheless possible in virtue of the “adequacy principle” as introduced in Sect. 2. To remember, Earman’s adequacy criteria establish, through physical symmetries (in particular, space–time symmetries), a correlation between, on the one hand, the dynamical structure of a theory and, on the other, its space–time structure. Hence, the adequacy criteria would entitle us to move from time-reversal symmetry at the level of description to the structure of time at the level of the basic ontology. In this way, the TRA can go through since time-reversal invariance entitles us to such an inference. It is important to note that this reply assumes an ‘ontic’ reading of the principles. We submit that Humeanism can block this reply by endorsing, instead, an ‘epistemic’ reading of Earman’s adequacy criteria.

Our argument is that the ontic reading should be discouraged since it entails that there must be a *metaphysical* connection between the structure of time and the dynamics. This requires a two-fold reification. It first requires that the structure of the dynamics of a theory does not just encode regularities in an abstract mathematical structure but captures *real* nomic relations (or nomic entities) in the basic ontology. Hence, any property we can ascribe to the dynamics is *ipso facto* a property of such objective nomic relations in the basic ontology as well as a property of any other structure correlated with them. Otherwise, it is not clear why we are entitled to read off features of the basic ontology from the fact that the dynamics of the theory is time-reversal invariant.

It is blatant that such a reading is at odds with Humeanism. For this reason, we prefer the epistemic reading. By holding that physical laws do not express primitive nomic relations among events or states of the world, it is no longer obvious that their symmetries can be straightforwardly connected with the structure of time in the Humean Mosaic. According to our view, the property of being time-reversal invariant belongs to the non-empirical virtues leading to a simplification of the dynamics instead of revealing a fundamental feature of the Humean Mosaic. Then, nature (the Mosaic) is not time-reversal invariant, but physical theories as axiomatic systems are. What Earman’s adequacy criteria then express is a formal connection between theoretical structures within the best system—the dynamical structure in the form of differential equations and the geometrical structure of the supporting space. Indeed, such principles are postulated in the aim of accounting for the Mosaic’s behavior while striving for the best balance between simplicity and informativeness. If this reading is endorsed, it is no longer clear why the correlation between the structure of the dynamics and the structure of time has any metaphysical consequences for time in the ontology.

For the sake of illustration, consider how determinism is treated in the best system account. In general, deterministic theories are much simpler than indeterministic ones. In a deterministic theory, given propositions that state the laws and initial or boundary conditions at an arbitrary time, it is possible to derive the propositions describing the entire past and future evolution of the objects of the theory. Hence, it is epistemically desirable to formulate physical theories in a deterministic manner when striving for the best system, because one thereby achieves an optimum of simplicity and informativeness. But it does not follow from this that the Mosaic is deterministic. On Humeanism, there is nothing that governs, predetermines, produces, or brings about the evolution of the Mosaic. That evolution simply happens. Consider the (apparent) conflict between free will and determinism: on Humeanism, deterministic physical theories cannot come into conflict with free will, because determinism is a feature of the theory and not of the ontology (see eg. Beebee & Mele, 2002).

To reinforce this Humean view of time-reversal invariance, it is worth noting that many space–time symmetries, time-reversal invariance among them, are frequently taken as rule-prescribing principles, stipulated a priori. Brading and Castellani (2003) explain how space–time symmetries have been increasingly conceived as guides to theory construction rather than as a consequence of a theory. Insofar as space–time symmetries are conceived of as rule-prescribing principles guiding theory construction, they fit well in the best system account as playing the heuristic role of overarching principles that help us find regularities and connections in the first-order generalizations. In the same vein, Arntzenius and Greaves (2009) advocate for working out the implementation of time reversal in classical electromagnetism by assuming (or stipulating) that the theory is time-reversal invariant (which they suggestively call the “text-book account”). This view of time-reversal invariance reinforces its heuristic role and discourages any reification. The Humean view of symmetries is then more naturalistic than its stronger realist alternatives since it has support in how today’s physics understands physical symmetries. Heuristic and epistemic principles guiding theory construction are not reliable means to obtain substantive claims about the basic ontology.

## Revisiting the TRA and conclusions

The main aim of the paper is to provide a Humean view of time-reversal symmetry that renders the TRA harmless. We did this by construing physical symmetries as second-order generalizations that seek to simplify, systematize, and unify first-order generalizations that supervene upon the behavior of local matter of facts in the Humean Mosaic. In this way, we make time-reversal symmetry a primarily epistemic property of the best system, and not a property of the basic ontology. When viewed from this Humean perspective, the TRA loses much of its persuasive force since it seems to assume a connection between (some) theoretical structures (i.e., the dynamical structure and the geometrical structure) and the basic ontology (i.e., the Humean Mosaic) that is illicit if Humeanism on symmetries is adopted. A reconstruction of the TRA in a Humean vein will prove it much less powerful than the original version.

To remind, the TRA states that:

- *P1* If the (fundamental) dynamical laws of physics are time-reversal invariant, then a primitive direction of time is metaphysically unnecessary.
- *P2* We should not posit unnecesary properties in our basic ontology.
- *P3* It happens that (most) laws of physics are time-reversal invariant.
- *P4* A primitive direction of time is then unnecesary.
- *C* We should not posit a primitive direction of time in the basic ontology.

It is easy to see that Humean symmetries do not entitle us to hold the conditional in *P1*. As previously noted, *P1* can only be true *if* some non-Humean view of law is endorsed. On Humeanism, we can accept that physical theories should be formulated in such a way that dynamical symmetries go hand-in-hand with space–time symmetries, and we can accept that time-reversal invariance is a crucial feature of the best systems; but this is just a heuristic, epistemic guide to theory construction, not a guide to ontology. *P3* just shows that most physical theories have been successful in achieving a time-reversal symmetric formulation. That is, they have been successful in exhibiting an epistemic virtue in the formulation of the first-order generalizations of the regularities they intend to describe. Since *P4* does not follow under this construal, the conclusion does not follow either.

To conclude, one of the main arguments against primitivism about the direction of time is the TRA. Under our Humean view of time-reversal symmetry, the TRA is rendered harmless. Therefore, Humean time-reversal symmetry is not a threat to the friends of a primitive direction of time. Of course, Humeanism can go together with rejecting a primitive direction of time (as it can go together with the rejection of free will): the basic ontology may display only variation in an eternal configuration of matter. But the advantages and disadvantages of this view should be assessed independently of time-reversal symmetry. The problem of whether physical theories are time-reversal invariant then boils down to whether it is epistemically and heuristically desirable to formulate our theories in a time-reversal invariant way. A failure of time-reversal invariance thus shows that some simplification, systematization, and unification had to be given up in order to deliver greater informativeness. But it has nothing to do with whether the Mosaic comes equipped with a primitive direction of time. Issues in theory construction should not be conflated with issues in ontology.

## Data Availability

Not applicable.

## Footnotes

1 This view contrasts with other Humean views, as that of Michael Hick’s (Hicks, 2019), where symmetries as considered of as generalizations of first-order facts. More on this below.

2 A canonical example of this is the formulation of Newtonian classical mechanics. If the Newtonian space–time has more structure than is required for the theory’s dynamical symmetries, then it is advisable to reformulate the theory as supported by less structured space–time, like the Galilean space–time.

3 Since physical symmetries come in many shapes and flavors, it would be tempting to think that such a plurality leads to some sort of ‘metaphysical’ pluralism, where some physical symmetries can be real, whereas others can be epistemic. We reject this view. Once again, an analogy with the metaphysical debates on laws will be instructive. Humeanism about laws does not defend that some laws are regularities, whereas others govern phenomena. Neither do defenders of the governing view hold that some laws govern, whereas others supervene upon dispositional properties. Our Humean approach to symmetries is similar –although we can accept that some form of methodological, or epistemic pluralism about symmetries might be useful, further argumentation is required to state that the factual plurality of symmetries that physics exhibits is a case of genuine metaphysical pluralism.

4 Fonda and Ghirardi (1970, ch. 1.7) actually distinguish two active views. In what they call the “first active view”, a symmetry $S$ changes the physical situation, $S:P\to \overline{P }$, and the observer, $S:O\to \overline{O }$, while in the “second active view”, a symmetry $S$ only changes the physical situation, $S:P\to \overline{P }$, which is described by the same observer $O$ in two different descriptions. For our argument, the distinction is not relevant and the second active view was adopted, since it is the most common in the literature.
